# Mechanisms of transmurally varying myocyte electromechanics in an integrated computational model

**DOI:** 10.1098/rsta.2008.0088

**Published:** 2008-07-01

**Authors:** Stuart G. Campbell, Sarah N. Flaim, Chae H. Leem, Andrew D. McCulloch

**Affiliations:** 1 Department of Bioengineering, University of California, San Diego9500 Gilman Drive no. 0412, La Jolla, CA 92093, USA; 2 Computing Laboratory, University of OxfordWolfson Building, Parks Road, Oxford OX1 3QD, UK; 3 Department of Physiology, University of Ulsan College of Medicine388-1 Poongnap-Dong Songpa-Ku, Seoul 138-736, South Korea

**Keywords:** transmural heterogeneity, cardiac electromechanics, computational modelling

## Abstract

The mechanical properties of myocardium vary across the transmural aspect of the left ventricular wall. Some of these functional heterogeneities may be related to differences in excitation–contraction coupling characteristics that have been observed in cells isolated from the epicardial, mid-myocardial and endocardial regions of the left ventricle of many species, including canine. Integrative models of coupled myocyte electromechanics are reviewed and used here to investigate sources of heterogeneous electromechanical behaviour in these cells. The simulations (i) illustrate a previously unrecognized role of the transient outward potassium current in mechanical function and (ii) suggest that there may also exist additional heterogeneities affecting crossbridge cycling rates in cells from different transmural regions.

## 1. Introduction

Although three-dimensional wall strains are highly inhomogeneous across the transmural aspect of the ventricular wall (Waldman *et al*. 1988), several studies showed that fibre strain distributions are relatively uniform during contraction (Costa *et al*. 1999; Tseng *et al*. 2000; Mazhari *et al*. 2001) owing to the effects of torsion and myofibre angle distributions (Arts *et al*. 1994). However, more recent studies using higher-resolution measurements have revealed significant spatial and temporal differences in myofibre strain in the left ventricular (LV) wall throughout the cardiac cycle (Ashikaga *et al*. 2004, 2007). Heterogeneities of structure, function and composition have been reported in myocardial tissue and in myocytes isolated from different regions in the LV wall. These heterogeneities are likely to contribute to complex transmural patterns of myofibre strain, though the precise nature of these relationships and their relevance to global heart function are not fully understood.

### (a) Transmural heterogeneity

Transmural heterogeneity of electrical activity and Ca^2+^ handling observed in tissue preparations (Wolk *et al*. 1999) can be explained largely by intrinsic differences in individual myocytes isolated from the endocardial, mid-myocardial and epicardial regions of the LV wall (Liu *et al*. 1993; Laurita *et al*. 2003). Action potential (AP) morphology is different among the three ventricular cell subtypes, with epi- and mid-myocardial cells showing a prominent phase 1 ‘notch’ that is essentially absent in endocardial cells (Liu *et al*. 1993). The magnitude of the notch is primarily determined by the transient outward K^+^ current (*I*_to1_), which is much smaller in endocardial cells (Litovsky & Antzelevitch 1988) as a result of transmural gradients in the expression of K^+^ channel interacting proteins (Rosati *et al*. 2003). AP duration (APD) has a significant transmural variation, with mid-myocardial cells exhibiting the longest APD (Liu *et al*. 1993). A transmural gradient in L-type Ca^2+^ channel current (*I*_CaL_) density was observed in one study, with endocardial cells showing higher values than epi- and mid-myocardial cells (Wang & Cohen 2003). This finding remains controversial, however, as several other studies showed no difference in *I*_CaL_ density among the three cell types (Li *et al*. 2002; Bányász *et al*. 2003; Cordeiro *et al*. 2004). Finally, the expression of sarcoplasmic reticulum Ca^2+^-ATPase (SERCA) is highest in epicardial cells and most probably explains the faster decay of the Ca^2+^ transient and increased sarcoplasmic reticulum (SR) load observed in these cells (Laurita *et al*. 2003; Cordeiro *et al*. 2004).

Fewer studies have focused on transmural variations in the passive and active mechanical properties of isolated myocytes, though some important differences have been documented. Transmural variation of passive myocyte stiffness has been observed in the rat heart and is associated with changes in overall titin expression levels (Cazorla *et al*. 2005). The ratio of titin isoform expression is also thought to modulate passive myocyte stiffness (Cazorla *et al*. 2000*a*), and the transmural gradients in this ratio have been observed in the canine LV (Bell *et al*. 2000). Cazorla *et al*. (2000*b*) identified distinct length–tension relationships between epicardial and endocardial cells isolated from rat and ferret hearts. Epicardial myocytes have been shown to reach peak unloaded shortening more quickly than those from the endocardium in both guinea-pig (Wan *et al*. 2003) and dog (Cordeiro *et al*. 2004).

Cordeiro *et al*. (2004) have conducted the only experimental study to date examining transmural heterogeneity of ionic currents, Ca^2+^ handling and mechanical function (electromechanics). They identified several key distinctions in the time course of unloaded shortening among canine LV epi-, mid- and endocardial cells. Endocardial cells displayed a comparatively longer delay in the onset of shortening, while epicardial cells showed the fastest shortening kinetics among the cell subtypes. Significantly, this study did not show any differences in *I*_CaL_ density or excitation–contraction (EC) coupling gain among the three myocyte subtypes. Furthermore, the amplitudes of the Ca^2+^ transient and the peak percentage of cell shortening were not seen to be significantly different among epi-, mid- and endocardial cells. The authors concluded that some, but not all, of the differences in shortening time course were related to AP morphology and Ca^2+^ handling, leaving room for additional, as yet unidentified, sources of mechanical heterogeneity.

Computational models of ventricular myocyte EC coupling have been useful for investigating the mechanisms of functional differences across the LV wall (Viswanathan *et al*. 1999; Solovyova *et al*. 2003; Saucerman *et al*. 2004; Flaim *et al*. 2006). With the exception of the work by Solovyova *et al*. (2003), none of these models includes the myofilament interactions responsible for cell shortening. Several of the heterogeneities mentioned above were mechanistically integrated into a recent model of canine EC coupling (Greenstein *et al*. 2006) by our group in the form of three different parameter sets, one for each myocyte subtype (Flaim *et al*. 2006). This model successfully reproduced several of the observations of Cordeiro *et al*. (2004), including differences in AP morphology and Ca^2+^ handling among cell subtypes.

### (b) Models of myocyte electromechanics

**Figure 1:**
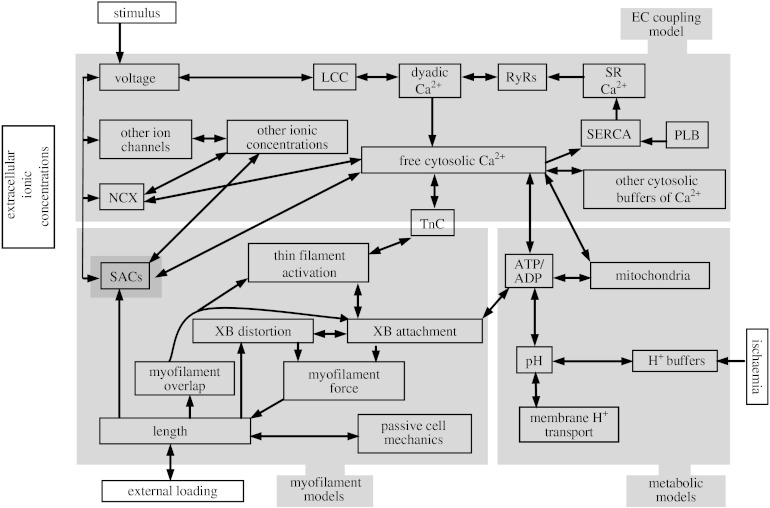
Diagram of generalized functional coupling and feedback mechanisms in the models of myocyte electromechanics. Entities that broadly couple cell processes include voltage, Ca^2+^, length, ATP/ADP and pH. Single arrows represent one-way coupling; double-headed arrows indicate two-way interaction between components. Interactions not shown for clarity include effects of pH on XB attachment, TnC, NCX, RyRs and SERCA; effects of length on RyRs; and effects of ATP on SERCA and NCX. Abbreviations: LCC, L-type Ca^2+^ channel; RyRs, ryanodine receptors; SERCA, sarcoplasmic reticulum Ca^2+^-ATPase; PLB, phospholamban; NCX, Na^+^/Ca^2+^ exchanger; SACs, stretch-activated channels; TnC, troponin C; XB, crossbridge.

Most models of myocyte electromechanics are combinations of previously published models of EC coupling subsystems, coupled via state variables including Ca^2+^, membrane potential, sarcomere length, pH and ATP. Figure 1 summarizes the mechanisms of coupling and feedback now represented in various system models of ventricular myocytes. While no single published model includes all these mechanisms, this figure demonstrates the feasibility of an integrated model of this type.

Modelling of ionic currents giving rise to the cardiac AP is the most mature of those subsystems involved in cellular electromechanics (reviewed in detail by Noble & Rudy 2001). Recent developments in the modelling of myocyte electrophysiology (EP) include continuous-time Markov models allowing for more mechanistic representations of ion channel behaviour individually (Irvine *et al*. 1999) and in the context of the whole cell (Clancy & Rudy 1999; Flaim *et al*. 2006). Detailed understanding of the events underlying Ca^2+^-induced Ca^2+^ release (CICR) is relatively recent, and thus models of the same have developed more slowly than those of myocyte EP (see review by Rice & Jafri 2001). The most advanced local control models of CICR (Greenstein & Winslow 2002) are too computationally demanding to be used in multiscale cardiac modelling. More recent models have used time-scale decomposition to capture important properties such as graded Ca^2+^ release while remaining computationally practicable for integration into larger-scale analyses (Hinch *et al*. 2004; Greenstein *et al*. 2006). Models of myofilament activation and force production have also been slow to mature owing to gaps that remain in the understanding of actin–myosin interactions and their role in thin filament activation (see review by Rice & de Tombe 2004). Recent myofilament models have addressed these deficiencies to some degree, and reproduce many experimentally observed characteristics of cardiac muscle contraction (Landesberg *et al*. 2000; Solovyova *et al*. 2002; Niederer *et al*. 2006; Rice *et al*. 2008).

Integrated models of myocyte electromechanics have been profitably used to investigate many whole-cell phenomena not within the grasp of smaller models. One of the first models to include several EC coupling mechanisms in equal detail was that of Rice *et al*. (2000), which was used to examine aspects of short-term interval–force relationships in cardiac muscle. Other subsequent models have been used to examine the relationship between force production and pacing interval (Matsuoka *et al*. 2003; Iribe *et al*. 2006). Iribe *et al*. (2006) used an integrated model of myocyte function to describe the role of Ca^2+^/calmodulin-dependent kinase II in producing Ca^2+^ transient alternans during the decay of post-extrasystolic potentiation.

Models of myocyte electromechanics have also been extensively used to investigate the dynamic relationship that exists between the length or tension of myocytes and their electrical activity. Acute alteration of the AP by changes in myocyte length (mechanoelectric feedback) has been modelled in a number of studies (Kohl *et al*. 1998; Kohl & Sachs 2001; Nickerson *et al*. 2001; Solovyova *et al*. 2004). Others have used integrated models to evaluate proposed mechanisms underlying the slow force response to stretch (Bluhm *et al*. 1998; Niederer & Smith 2007).

Other models have added components such as mitochondrial metabolism and Ca^2+^ uptake (Cortassa *et al*. 2006) and pH regulation (Crampin & Smith 2006) to fully coupled myocyte models. These modifications enabled analysis of metabolic control in the heart under normal conditions (Cortassa *et al*. 2006) and of the mechanisms by which acidosis affects myocyte contractility during ischaemia (Crampin & Smith 2006).

Here, we describe our use of a coupled computational model of electromechanics to investigate mechanisms underlying transmural variations in myocyte function in the canine LV. We coupled recent models of canine EC coupling (Flaim *et al*. 2006) and myofilament force production (Rice *et al*. 2008) in order to test the hypothesis that known heterogeneities in AP morphology and Ca^2+^ transients are sufficient to explain experimentally observed differences in the time courses of unloaded cell shortening. The analysis suggests an important role for transmural variations in *I*_to1_ in determining regional shortening and also indicates that variations in crossbridge function may be present.

## 2. Methods

### (a) Model of myocyte electromechanics

We started with the endocardial canine myocyte model formulated by Flaim *et al*. (2006) and coupled it to the recent model of myofilament force production and cell mechanics of Rice *et al*. (2008). Forward coupling between EP/Ca^2+^ handling and myofilaments was accomplished by driving the myofilament model with the variable representing [Ca^2+^]_i_. Reverse coupling was achieved by replacing the static Ca^2+^ buffering by TnC low-affinity regulatory sites used in the model of Flaim *et al*. (2006) with the dynamic flux of Ca^2+^ on and off the myofilaments described by the Rice model. The modified differential equation describing [Ca^2+^]_i_ as well as other details of model coupling can be found in the electronic supplementary material.

The complete electromechanics model was programmed in Matlab and run for more than 50 beats to achieve steady-state conditions for each parameter set used. A basic cycle length (BCL) of 2000 ms was used for all simulations to allow valid comparison with the experiments of Cordeiro *et al*. (2004).

AP-clamp protocols were simulated by prescribing the membrane voltage with an appropriate time-varying function rather than allowing it to be determined by membrane currents. The epicardial and endocardial AP waveforms used in AP-clamp simulations were digitized from representative traces reported by Cordeiro *et al*. (2004).

### (b) Modification of published model parameters

All adjustments to model parameters from those originally published are presented in the electronic supplementary material. These adjustments were intentionally kept to a minimum, and were only made to correct the largest differences between experimental data and model behaviour. EP/Ca^2+^ handling parameter sets for epi-, mid- and endocardial cells set by Flaim *et al*. (2006) were retained, along with those described in the original canine EC coupling model of Greenstein *et al*. (2006). The lone exception was an increase in the number of Ca^2+^ release units from 50 000 to 75 000 to increase the amplitude of the Ca^2+^ transient at a BCL of 2000 ms to more typically observed levels. This same parameter value has been used by others (Niederer & Smith 2007). A few minor changes to the original myofilament model parameters (Rice *et al*. 2008) were made to increase the stability of unloaded shortening responses in the coupled electromechanics model. Alterations to parameters governing myofilament Ca^2+^ sensitivity and cooperativity were also made in order to give a time course and magnitude of shortening that agreed qualitatively with experiments (Cordeiro *et al*. 2004). These and other adjustments to myofilament model parameters described throughout the remainder of this paper are listed in detail in the electronic supplementary material.

### (c) Modelling of the exogenous Ca^2+^ buffer fluo-3

We represented the buffering of intracellular Ca^2+^ by fluo-3 dye using a standard differential equation for simple buffering (equation (S2) in the electronic supplementary material). Ca^2+^ transients measured experimentally reflect the time course of Ca^2+^ bound to the indicator molecule ([Ca^2+^]_fluo_) and are often calibrated to correspond to the concentration of cytosolic Ca^2+^. To mimic this measurement, the model reports the time course of [Ca^2+^]_fluo_ scaled to vary between the minimum and maximum values of [Ca^2+^]_i_. We have called this quantity [Ca^2+^]_i/app_, the apparent cytosolic Ca^2+^ concentration. This modification enables direct comparison of model output with experimental measurements.

In all simulations where fluo-3 was present, the concentration of this indicator in the cytosol was assumed to be 15 μM, corresponding to the concentration of fluo-3 used in bathing solutions by Cordeiro *et al*. (2004). New steady-state conditions for the coupled model had to be found when fluo-3 was included, as the buffering action of this indicator was substantial enough to alter SR load and other model values. This was accomplished as before by running the model for more than 50 beats in the presence of 15 μM fluo-3.

### (d) Simulation of shortening under clamped [Ca^2+^] and Ca^2+^ flux conditions

The myofilament model of Rice *et al*. (2008) was used independently of the coupled electromechanics model to study Ca^2+^ contraction dynamics under various conditions. Two Ca^2+^ input modes were used in these simulations. The first allows myocyte contraction and shortening to be driven with a prescribed Ca^2+^ transient, which automatically sources as much Ca^2+^ flux as needed to maintain the command [Ca^2+^]_i_ signal. This mode was used to study the shortening response to specific Ca^2+^ transients.

A second type of Ca^2+^ clamp was created whereby a fixed Ca^2+^ flux, *J*_clamp_, was used to drive shortening. This approach is similar to the one originally used to estimate Ca^2+^ flux across the SR (Sipido & Wier 1991). A differential equation describing the time course of [Ca^2+^]_i_ was added to the original myofilament model equations in which d[Ca^2+^]_i_/d*t* is set equal to the sum of different Ca^2+^ fluxes,

(2.1) $$\frac{\text{d}{\left[{\text{Ca}}^{2+}\right]}_{\text{i}}(t)}{\text{d}t}=J_{\text{clamp}}(t)+J_{\text{fluo}}\left({\left[{\text{Ca}}^{2+}\right]}_{\text{i}},t\right)+J_{\text{TnC}}\left({\left[{\text{Ca}}^{2+}\right]}_{\text{i}},t\right)+J_{\text{SERCA}}\left({\left[{\text{Ca}}^{2+}\right]}_{\text{i}},t\right),$$

where *J*_fluo_, *J*_TnC_ and *J*_SERCA_ are [Ca^2+^]_i_ and time-dependent Ca^2+^ fluxes describing buffering by fluo-3, buffering by TnC and uptake by SERCA, respectively. The fluxes representing the buffering of Ca^2+^ by fluo-3 and TnC are calculated as described above and in the original work of Rice *et al*. (2008), while uptake of SERCA is modelled as in the EP model with the assumption that SR content is fixed (see electronic supplementary material). This ‘flux-clamp’ mode allows the Ca^2+^ transient driving contraction to be flexibly determined under changing conditions such as the presence and absence of fluo-3.

## 3. Results

### (a) Model validation

**Figure 2:**
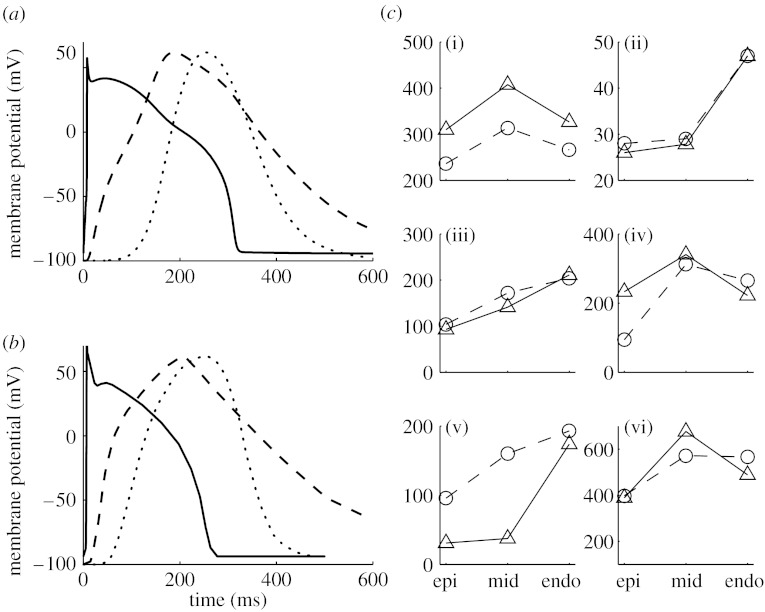
Comparison of simulated and experimentally measured canine ventricular myocyte electromechanics. (*a*) Simulated model and (*b*) experimentally measured time courses of endocardial myocyte membrane potential (solid curves), [Ca^2+^]_i/app_ (dashed curves, see §2 for definition) and unloaded cell shortening (dotted curves). [Ca^2+^]_i/app_ and shortening traces have been scaled and are displayed in arbitrary units to facilitate comparison. (*c*) Comparison of model (triangles) and experimentally reported (circles) transmural trends in EC coupling. (All experimental traces and values are taken from Cordeiro *et al*. 2004.) (i) APD (ms); (ii) Sh latency (ms); (iii) Sh TTP (ms); (iv) RT_90_ (ms); (v) CaT TTP (ms); (vi) CaT RT_90_ (ms). Abbreviations: Sh, shortening; CaT, Ca^2+^ transient; TTP, time to peak; RT_90_, time from peak to 90 per cent relaxation.

The fully coupled electromechanical model with endocardial myocyte parameters produced a time course of EC coupling events comparable with those reported experimentally (figure 2*a*,*b*). The model displayed unloaded cell shortening characteristics of latency to onset, time to peak and peak percentage of shortening that agreed with mean experimental values to within the reported standard deviations (Cordeiro *et al*. 2004). The model showed qualitative agreement with other experimentally reported values. Model time from peak of unloaded shortening to 90 per cent relaxation (RT_90_) was 222 ms, compared with a mean value of 265 ms (Cordeiro *et al*. 2004). Modelled Ca^2+^ transients (time course of Ca^2+^ bound to fluo-3 and scaled to peak free cytosolic Ca^2+^) showed a latency to the onset of 16 ms (versus 17.4 ms experimental mean), time to peak of 161 ms (versus 193 ms), RT_90_ of 489 ms (versus 566 ms) and peak cytosolic Ca^2+^ concentration of 580 nM (versus 663 nM). The APD of the endocardial model was 326 ms, compared with a mean value of 266 ms reported in experiments at the same BCL of 2000 ms (Cordeiro *et al*. 2004).

### (b) Transmural heterogeneity in a model of myocyte electromechanics

Parameter sets describing heterogeneous properties of membrane currents and Ca^2+^ handling for epicardial and mid-myocardial cells were proposed by Flaim *et al*. (2006). We performed simulations of unloaded shortening using these parameter sets in the electromechanics model to evaluate the potential for these proposed differences to explain distinctions in mechanical function reported by Cordeiro *et al*. (2004).

The transmural trend in APD achieved in the original model (Flaim *et al*. 2006) was retained in the electromechanics model and agrees with Cordeiro *et al*. (2004) and other reports (Liu *et al*. 1993). Trends in cell mechanical function between the three cell types agreed qualitatively with those reported experimentally, including (i) longer latency to the onset of contraction in endocardial cells, (ii) shorter time to peak shortening in epicardial cells and (iii) longer RT_90_ in mid-myocardial cells (figure 2*c*). Peak percentage cell shortening matched measurements within the reported standard deviation for all three cell types (not shown), indicating a reasonable relation between peak systolic [Ca^2+^]_i_ and myofilament Ca^2+^ sensitivity.

Conversely, the model failed to reproduce the significantly reduced RT_90_ observed experimentally in epicardial cells compared with the other cell types (figure 2*c*). The epicardial and mid-myocardial parameter sets both yielded Ca^2+^ transients with a rapid time to peak that was not fully consistent with measured representative traces or mean values (Cordeiro *et al*. 2004). The mid-myocardial cell model also produced a Ca^2+^ transient RT_90_ of approximately 200 ms longer than that of the endocardial model, while this difference was not seen in experiments.

### (c) AP-clamp simulations

Since the model showed a strong dependence of SR Ca^2+^ release on the AP waveform in epicardial and mid-myocardial cells, the influence of AP morphology on Ca^2+^ release and shortening was examined in the absence of other differences (such as SR load) using AP-clamp conditions. Initial conditions for all model state variables were taken from the mid-myocardial model run to steady state, after which a single beat was elicited in response to a representative endocardial or epicardial AP digitized from the literature (Cordeiro *et al*. 2004).

**Figure 3:**
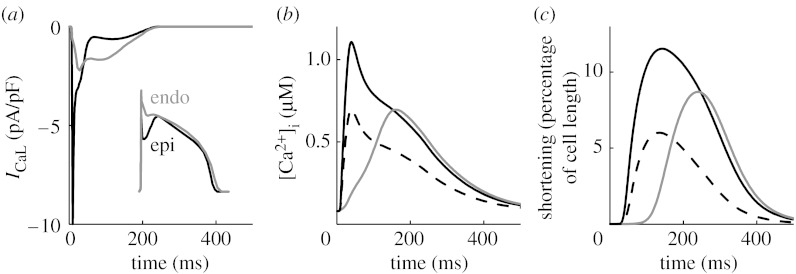
Influence of AP morphology on (*a*) *I*_CaL_, (*b*) Ca^2+^ transient and (*c*) unloaded shortening. Results shown are simulated responses to typical epi- or endocardial APs (inset to (*a*), digitized from Cordeiro *et al*. 2004) in the electromechanics model. Solid and shaded curves correspond to epi- and endocardial AP-clamp, respectively. Dashed curve in (*c*) shows unloaded shortening by the model in response to a clamped Ca^2+^ transient shown in (*b*) as the dashed curve (see text for details).

L-type Ca^2+^ channel currents (*I*_CaL_), Ca^2+^ transients and cell shortening in response to epicardial and endocardial AP waveforms showed large differences in amplitude and time course (figure 3). The epicardial AP generated a peak current five times greater than that of the endocardial AP. By contrast, the endocardial AP produced a marked prolongation of the secondary phase of *I*_CaL_, though the respective currents ultimately terminated at about the same time, around 230 ms.

Differences in *I*_CaL_ had a clear effect on downstream EC coupling events. The peak of the Ca^2+^ transient under epicardial AP-clamp arrived approximately 120 ms faster and was approximately 40 per cent larger than that of the endocardial AP (figure 3*b*), corresponding to the greater amount of triggering Ca^2+^ in the initial phase of *I*_CaL_. The endocardial AP-elicited Ca^2+^ transient reached a peak magnitude above 50 per cent of that of the other transient, despite the fivefold smaller initial trigger current through a longer secondary phase of *I*_CaL_ (figure 3*a*, shaded curve). The onset of unloaded shortening was plainly different between the two cases, with shortening in response to the endocardial AP starting approximately 30 ms after that of the epicardial AP (figure 3*c*). Peak unloaded cell shortening in response to the epicardial AP was reached approximately 100 ms faster than that observed under endocardial AP, and was approximately 30 per cent greater in magnitude.

To understand better the relationship between Ca^2+^ transient time course and unloaded shortening dynamics, we simulated an unloaded shortening event under a specified or ‘clamped’ Ca^2+^ transient corresponding to a scaled version of the epicardial AP-elicited transient (figure 3*b*, dashed curve). The transient was scaled such that its peak corresponded in magnitude with that of the endocardial transient in an attempt to isolate the effects of transient shape from those of absolute magnitude. Unloaded shortening during Ca^2+^ clamp (figure 3*c*, dashed curve) displayed shortening latency, time to peak and time from peak to 90 per cent relaxation all well within 10 per cent of those same values in epicardial AP-driven shortening (figure 3*c*, solid curve), showing that the time course of the Ca^2+^ transient was the primary determinant of shortening dynamics on this scale. Surprisingly, the contraction driven by the scaled epicardial Ca^2+^ transient resulted in a 30 per cent reduction in peak shortening compared with that from the endocardial AP-clamp, in spite of sharing the same absolute magnitude of peak cytosolic Ca^2+^.

### (d) Influence of an exogenous buffer on Ca^2+^ transients and unloaded shortening

Discrepancies between model-predicted and experimentally observed Ca^2+^ transients for mid-myocardial and epicardial cells limited the ability of the electromechanics model to discern potential sources of transmural heterogeneity downstream of Ca^2+^ handling. We therefore simulated unloaded shortening driven directly by representative Ca^2+^ transients measured experimentally in the three cell types.

**Figure 4:**
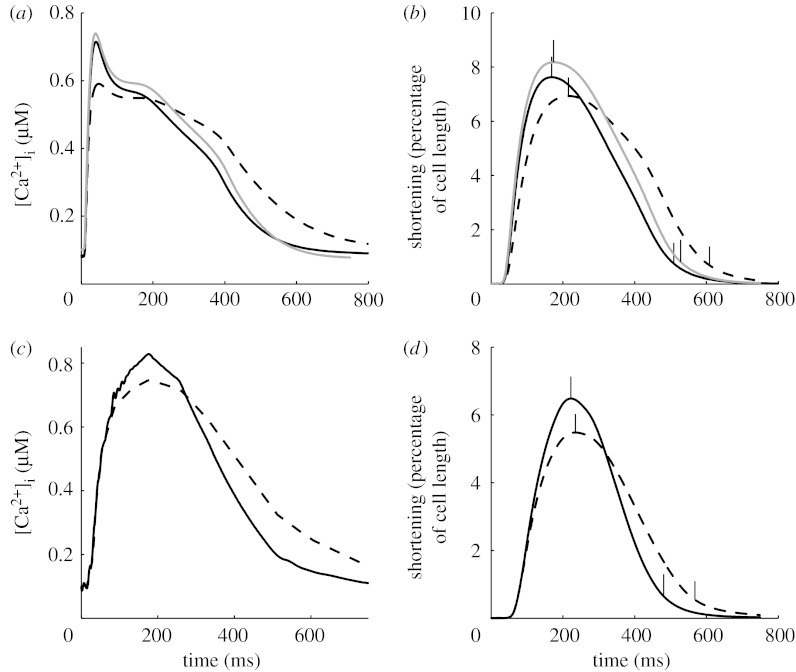
Effect of fluo-3 on the apparent Ca^2+^ transient and potential errors in the prediction of shortening in response to [Ca^2+^]_i/app_. (*a*) [Ca^2+^]_i_ (solid curve) taken from the mid-myocardial cell model with no fluo-3 present plotted with [Ca^2+^]_i/app_ (dashed curve) from the same model in the presence of 15 μM fluo-3. (*b*) Simulated cell shortening in response to the respective Ca^2+^ signals shown in (*a*), e.g. solid curve is shortening in response to the solid curve in (*a*) and so on. Shaded curves in (*a*) and (*b*) correspond to flux-clamp validation as described in the text. (*c*) This is similar to (*a*) but now compares a calibrated fluo-3 fluorescence signal (dashed curve, digitized from Cordeiro *et al*. 2004) with [Ca^2+^]_i_ predicted via the flux-clamp protocol (solid curve). (*d*) Shortening in response to the respective Ca^2+^ inputs shown in (*c*). Tick marks on curves in (*b*) and (*d*) indicate peak shortening and 90 per cent relaxation.

Before doing this, we added a simple representation of fluo-3 buffering and kinetics to the electromechanics model (as described in §2) to assess its effects on the Ca^2+^ transient and shortening. The concentration of fluo-3 was set to 15 μM, consistent with the concentration used in experiments (Cordeiro *et al*. 2004), and the model was run to steady state. We then compared [Ca^2+^]_i/app_ with [Ca^2+^]_i_ from the model containing no fluo-3 (figure 4*a*). Addition of the fluo-3 had negligible impact on the time to peak Ca^2+^, but lowered the magnitude of the peak by approximately 20 per cent through buffering of free Ca^2+^. Time from peak to 90 per cent decay of [Ca^2+^]_i/app_ was increased by 40 per cent over the same value calculated from [Ca^2+^]_i_. This prolongation of the Ca^2+^ transient is caused by competition for free Ca^2+^ between the high-affinity indicator and the usual Ca^2+^ uptake pathways, but also possesses an apparent component that reflects the dissociation kinetics of the dye. Overall, this simulation suggests that the apparent Ca^2+^ transient as assessed by indicator fluorescence is blunted at its peak and decays more slowly in comparison with the actual transient in the absence of this exogenous buffer.

To assess the type and magnitude of errors that could be introduced by driving cell shortening directly with measured indicator fluorescence, [Ca^2+^]_i/app_ (figure 4*a*, dashed curve) was used to drive shortening in the myofilament model (figure 4*b*, dashed curve). Peak shortening was reduced by approximately 10 per cent, while the time to peak shortening and RT_90_ were increased by 32 and 15 per cent over original values, respectively. These differences indicated the need for a method to estimate the true time course of intracellular Ca^2+^ from fluorescence measurements.

### (e) Flux-clamp validation

The electromechanics model was used to generate a test case for the predictive function of the flux-clamp input mode. An input flux, *J*_clamp_, was determined from the electromechanics model run to steady state in the presence of 15 μM fluo-3, and then used to drive the flux-clamp myofilament model in the absence of fluo-3. The resulting Ca^2+^ transient and unloaded shortening (figure 4*a*,*b*, shaded curves) were compared with traces from the base model. The predicted Ca^2+^ transient was close in magnitude and shape to that of the original simulation, and unloaded shortening time course showed only slight differences (less than 8% change from baseline for magnitude of shortening, time to peak and RT_90_).

### (f) Shortening responses to realistic Ca^2+^ transients

We used the flux-clamp protocol to estimate the true Ca^2+^ transient from a representative time course of indicator fluorescence from a mid-myocardial cell (Cordeiro *et al*. 2004). Differences similar to those seen in simulations using the fully coupled model (figure 4*a*) can be observed between experimentally measured [Ca^2+^]_i/app_ and [Ca^2+^]_i_ estimated using flux-clamp (figure 4*c*). These included increased magnitude and faster relaxation for the estimated transient. The estimated shortening in the absence of fluo-3 generated with the flux-clamped model (figure 4*d*, solid curve) was compared with shortening driven directly with the fluorescence data (figure 4*d*, dashed curve) using the same myofilament model parameters in both cases. The contraction driven by fluorescence displayed differences in magnitude of shortening (−15%), time to peak (+7.6%) and RT_90_ (+28.5%) compared with flux-clamp shortening.

**Figure 5:**
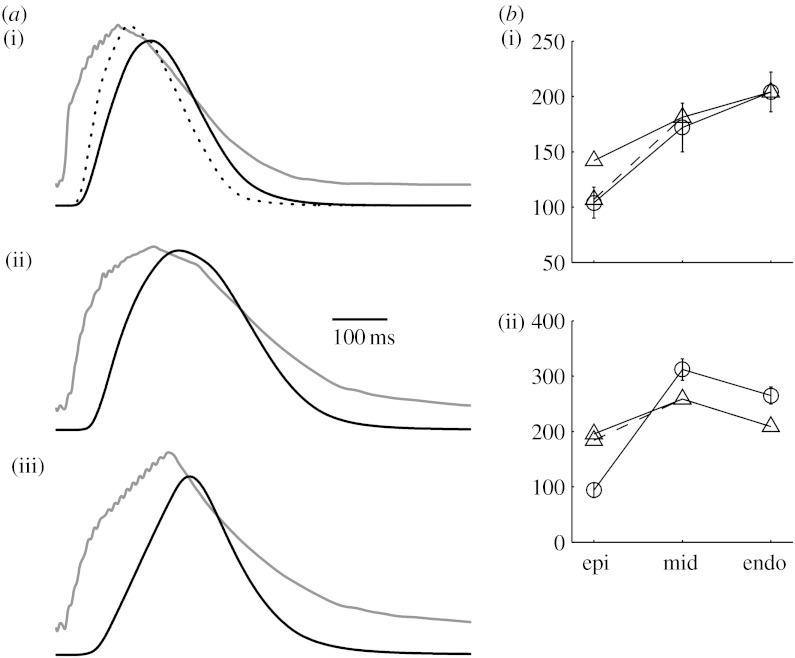
Simulated unloaded shortening for epi-, mid- and endocardial cells in response to measured Ca^2+^ transients corrected via flux-clamp protocol. (*a*) Time course comparison of [Ca^2+^]_i_ (shaded curves) and resulting cell shortening (solid curves) in each of the three cell types: (i) epi-, (ii) mid- and (iii) endocardial. The dotted curve shows shortening after an increase in crossbridge cycling rate consistent with the expression of the V1 myosin isoform (see text for details). (*b*) Comparison of simulated (i) shortening TTP (triangles) and (ii) RT_90_ (triangles) with mean values reported by Cordeiro *et al*. (2004; circles) for the three cell types. The dashed lines leading to triangles were determined from the dotted curve in (*a*) and once again represent the effect of increased crossbridge cycling rates.

The parameters of the myofilament model were adjusted until time to peak shortening and shortening magnitude under mid-myocardial cell flux-clamp were within the standard deviations of measured mean values (Cordeiro *et al*. 2004). The adjusted parameter set corresponded to a steady-state force–Ca^2+^ relationship possessing a half-activation value (Ca_50_) and Hill coefficient (*n*_H_) of approximately 1.0 μM and 3.5, respectively. Latency to the onset of shortening using these parameters was 12 ms slower than the experimentally reported mean; however, this may be attributed to noise in the original fluorescent signal or error in digitizing the trace, as even small differences in the initial rising phase of the Ca^2+^ transient have a strong effect on shortening latency. Additionally, RT_90_ was 54 ms shorter than the experimentally reported mean of 312 ms. This is most probably due to a secondary, slower time constant of relaxation seen in the representative shortening traces for mid- and endocardial cells (cf. figure 1 in Cordeiro *et al*. 2004), which the model did not reproduce. Retaining the myofilament model parameter set used to fit reported mid-myocardial cell shortening, the flux-clamp protocol was used to determine driving fluxes matching representative epicardial and endocardial fluorescence traces, and unloaded shortening in the absence of indicator was predicted as previously (figure 5*a*). The myofilament model parameters adjusted to match the mid-myocardial shortening response also resulted in good reproduction of reported values for endocardial cells (figure 5*b*). Shortening RT_90_ for the mid- and endocardial cell responses showed the same systematic deviation from reported mean values and displayed the correct transmural trend (figure 5*b*). Peak cell shortening under endocardial flux-clamp was within the experimentally reported variation for this value (not shown).

Shortening simulated using the base myofilament model parameter set in response to epicardial flux-clamp did not show good agreement with experimental values of time to peak and RT_90_ (figure 5*b*). Time to peak shortening and RT_90_ were both slower than experimental means by 38 and 101 ms, respectively. These results were strongly suggestive of an underlying difference in myofilament properties between epicardial cells and the other two cell subtypes.

### (g) Testing hypothesized myofilament heterogeneities

**Figure 6:**
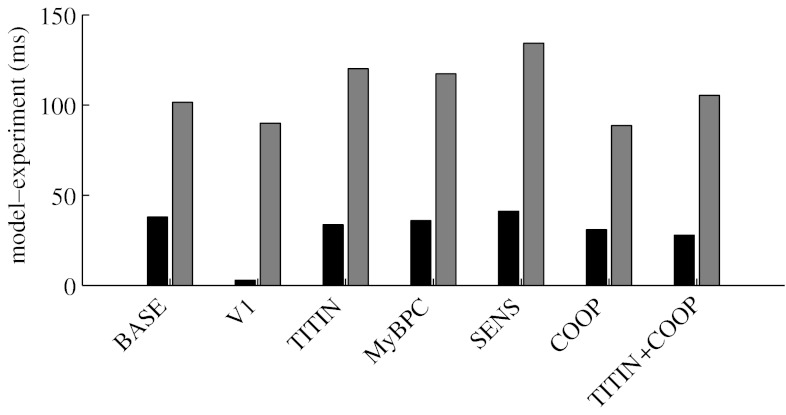
Testing of hypothesized mechanisms of altered Ca^2+^ contraction coupling in epicardial myocytes. Description of the hypothesis represented by each abbreviation is presented in the text. Other abbreviations are the same as given in the legend of figure 2. Black bars, Sh TTP; grey bars, Sh RT_90_.

We considered and quantitatively tested several different hypotheses regarding changes to the myofilaments that might reconcile the discrepancies in Ca^2+^ contraction dynamics between the model and experimental measurements in epicardial cells. As many of the proposed changes affected myofilament Ca^2+^ sensitivity, the flux-clamp protocol had to be repeated for each one in order to fit a new driving flux. Figure 6 shows the difference between model and experimental values of shortening time to peak and RT_90_ for each hypothesis tested in comparison with the original parameter set (BASE in figure 6). We tested the potential effects of transmural variation in myosin isoform expression (suggested by Cordeiro *et al*. 2004) by changing the crossbridge kinetic rates in the myofilament model to those described by Rice *et al*. (2008) as corresponding to the faster V1 myosin isoenzyme (V1 in figure 6). Passive cell stiffness was increased (TITIN in figure 6), consistent with increased expression of the stiffer N2B titin isoform observed in the canine epicardium (Bell *et al*. 2000). We hypothesized that phosphorylation of myosin-binding protein C, which is thought to increase crossbridge attachment rate (Stelzer *et al*. 2006), could be altered in epicardial cells (MyBPC in figure 6). We also tested the sensitivity of the model to increased myofilament Ca^2+^ sensitivity (SENS), increased myofilament cooperativity (COOP) and a combination of increased titin-based stiffness with steeper cooperative activation (TITIN+COOP). The myofilament model parameter sets used to represent each of these hypotheses are shown in the electronic supplementary material.

Of all the hypotheses tested in the model, none was able to account completely for the differences between the epicardial cell model and experimentally reported values (figure 6). The V1 and COOP parameter sets reduced RT_90_ below that shown by the BASE model, but not significantly. Overall, the relaxation of shortening in the model was not sensitive to any of the parameters changed in this process. Time to peak shortening was reduced to some extent by all but the SENS parameter set, which increased time to peak. Time to peak was most sensitive to the V1 parameter set, which brought this value to within the experimentally observed variation (figure 5*a*, dotted curve; figure 5*b*, dashed line and triangle).

## 4. Discussion

The present work suggests that much of the mechanical heterogeneity observed by Cordeiro *et al*. (2004) in canine myocytes may be explained by previously identified and modelled differences in EC coupling and clarifies the mechanisms by which this control is likely to operate. We have presented evidence that, where heterogeneity of Ca^2+^ transients does not completely explain differences in shortening, a specific alteration in myofilament protein expression reconciles experimental and modelling results.

### (a) Modulation of contractile dynamics by early repolarization and I_to1_

Cordeiro *et al*. (2004) reported significantly prolonged time between stimulus and the onset of unloaded cell shortening in endocardial cells in comparison with mid- and epicardial cells. In the coupled model, this was the result of a large difference in the slope of the rising phase of the Ca^2+^ transient. Early repolarization of the AP was seen as the primary modulator of this aspect of Ca^2+^ morphology (figure 3). As *I*_to1_ magnitude determines distinctions in early repolarization (Litovsky & Antzelevitch 1988), we conclude that it ultimately controls contraction latency to a large extent.

The process by which *I*_to1_ changes the time course of contraction is mechanistically explained by the electromechanics model. The slowed rate of early repolarization under endocardial AP-clamp allows less extracellular Ca^2+^ into the dyadic subspace, and results in only a small amount of Ca^2+^ release through the RyRs. However, a lower concentration of Ca^2+^ in the dyadic subspace causes Ca^2+^-dependent inactivation of L-type Ca^2+^ channels to proceed at a slower rate, prolonging the secondary phase of *I*_CaL_ relative to that seen under epicardial AP-clamp. The sustained nature of this phase causes enough SR Ca^2+^ release to generate a cytosolic transient that is delayed but still of significant amplitude. A similar relationship between the rate of early repolarization and Ca^2+^ transient shape has been seen experimentally (Sah *et al*. 2002).

Given the steep force–Ca^2+^ relationship of cardiac muscle, it seems counterintuitive that the magnitudes of shortening between the two AP-clamp cases would be closer than those of their respective Ca^2+^ transients. Simulations using realistic myofilament cooperativity reveal that the extent of unloaded shortening is strongly affected by the shape of the Ca^2+^ transient, not just the absolute magnitude (figure 3*b*,*c*). Cooperative recruitment of force slows the rate of force development at submaximal levels of myofilament activation (Campbell 1997), meaning that rapidly peaking Ca^2+^ transients allow less time for the system to reach maximal force than their slower counterparts. Steep cooperativity of myofilament force production may therefore function as a mechanism to preserve contractile function in endocardial cells in the absence of *I*_to1_. Similarly, Ca^2+^ transient morphology may explain why endocardial cells showed slightly larger peak unloaded shortening than the other cell types in spite of no significant difference in Ca^2+^ transient amplitude (Cordeiro *et al*. 2004).

### (b) Transmural heterogeneity of myofilament protein expression

Flux-clamp simulations suggested that mid- and epicardial cells could not share the same Ca^2+^ contraction dynamics. The increased SR Ca^2+^ content observed in epicardial cells (Cordeiro *et al*. 2004), probably due to increased SERCA expression (Laurita *et al*. 2003), may be responsible for faster time to peak of the Ca^2+^ transient compared with mid-myocardial cells. Increased SERCA also has the effect of speeding decay and (to a lesser extent) the time to peak of the Ca^2+^ transient. Yet the combined effects of *I*_to1_, increased SR content and increased SR reuptake of Ca^2+^, manifest in the experimentally measured epicardial Ca^2+^ transient, appear to be insufficient to account for the observed increase in contraction speed.

We showed using the model that increasing crossbridge kinetics in a manner consistent with increased expression of the faster V1 isoform of myosin produces a relationship between time course of free Ca^2+^ and time to peak shortening, which agreed well with experiments. This change alone was the most effective compared with several alternative hypotheses and suggests a transmural gradient in the expression ratio of V1 to V3 myosin, with V1 expression highest at the epicardium. Although no study of which we are aware has examined transmural gradients of myosin isoform expression in canine, others have been performed in rat (Carnes *et al*. 2004), in rabbit (Litten *et al*. 1985) and most recently in pig (Stelzer *et al*. 2008). These studies universally report higher expression of V1 myosin on the epicardium than endocardium, supporting our hypothesis.

We were unable to find a parameter set for the myofilament model of Rice *et al*. (2008) that would completely describe the epicardial myocyte Ca^2+^ contraction dynamics reported experimentally (Cordeiro *et al*. 2004). While speeding the crossbridge cycling rate allowed the model to correctly predict time to peak shortening, relaxation still proceeded at too slow a rate. Indeed, none of the parameter modifications we attempted was able to account for the faster relaxation rate reported experimentally for epicardial cells. This is not a general failure of the model, as the relaxation–Ca^2+^ relationship is apparently well reproduced in mid- and endocardial cells. Instead, this points to the absence of some mechanism not included in the model whereby faster crossbridge cycling influences deactivation of the thin filament.

### (c) Insights from tight coupling between EP and myofilament models

Modelling of cardiac EC coupling usually stops short of including contraction. Models and experiments have already examined the relationship between early repolarization (primarily via *I*_to1_) and the Ca^2+^ transient (Sah *et al*. 2002; Greenstein *et al*. 2006). These studies have emphasized the adverse effects of reduced *I*_to1_ on contraction seen in the diseased myocardium. However, it is clear that heterogeneous *I*_to1_ expression is present in the normal heart with little effect on the strength of contraction (Cordeiro *et al*. 2004). The electromechanics model suggests that several mechanisms working together can compensate for lower initial *I*_CaL_ in endocardial cells to preserve contractile function. Coupling of the EP and myofilament models allowed us to elucidate this subtle behaviour.

The majority of models of myocyte EC coupling assume a constant affinity of TnC for Ca^2+^, while experiments suggest that thin filament activation and crossbridge binding alter TnC buffering capacity (Davis *et al*. 2007). Owing to the steep cooperative regulation of crossbridge binding by Ca^2+^, the buffering capacity of the myofilament system may also be viewed as cooperative, undergoing large time- and Ca^2+^-dependent shifts within a single cardiac cycle. In the coupled electromechanics model, this dynamic buffering shortened APD relative to the original EC coupling model with static TnC buffering by reducing the buffering capacity of the myofilaments during the diastolic interval and allowing more rapid decline of [Ca^2+^]_i_ via SR uptake and extrusion through other mechanisms. Dynamic buffering of Ca^2+^ by the myofilaments played an especially important role in the flux-clamp protocol we had designed to estimate the Ca^2+^ transient after removal of the fluorescent indicator. In the absence of the exogenous buffer, the myofilament buffering capacity adjusted itself in a complex nonlinear manner, resulting in changes to Ca^2+^ transient morphology.

Most models of myofilament force production are tested using a clamped Ca^2+^ transient based on the time course of fluorescent indicators. We used the electromechanics model to show that such indicators can cause large errors in Ca^2+^ transient shape. Thus aspects of the EP/Ca^2+^ handling model allowed us to make greater quantitative use of the myofilament model. In summary, including tightly coupled models of all three subsystems improved the quality of all models involved.

### (d) Limitations

The models presented here have several limitations. Each of the published models that form the components of the present model contains limitations of its own (Flaim *et al*. 2006; Greenstein *et al*. 2006; Rice *et al*. 2008). The most important of these is the strong cycle-length dependence of Ca^2+^ transient time to peak in the Greenstein model (cf. fig. 6*b* in Greenstein *et al*. 2006), especially when using the parameter sets for mid- and epicardial cells presented by Flaim *et al*. (2006). This dependence caused the electromechanics model to produce qualitatively different Ca^2+^ transients unlike those reported experimentally when run at a matching cycle length of 2000 ms. This discrepancy did not affect the use of the model in elucidating the modulation of contraction time course by *I*_to1_, but did limit the ability of the model to identify potential heterogeneities of myofilament properties. This shortcoming was effectively overcome via the flux-clamp simulations, which allowed the prediction of shortening directly from experimentally reported Ca^2+^ transients.

We did not fully explore the effects of increased SERCA and higher SR Ca^2+^ load reported in canine epicardial cells (Laurita *et al*. 2003; Cordeiro *et al*. 2004). The model of Flaim *et al*. (2006) exhibits a higher SR Ca^2+^ load in epicardial cells relative to the other types at a cycle length of 1000 ms, which is consistent with experimental results. However, the electromechanics model at slower cycle lengths produced only a moderately elevated SR load, once again indicating an incorrect frequency dependence of Ca^2+^ handling. The result achieved in the model of Flaim *et al*. (2006) came by doubling the activity of SERCA relative to the other cell types to reflect the experimental reports of much higher SERCA expression in epicardial cells. When this hypothesis was implemented in similar fashion in the flux-clamp protocol, it resulted in unreasonable responses after withdrawal of fluo-3. As a result, we left SERCA parameters unchanged during epicardial cell flux-clamp and allowed any additional uptake of Ca^2+^ by SERCA present in these cells to be implicitly incorporated into the driving flux during the fitting process.

The flux-clamp protocol involves several assumptions. The cytosolic concentration of fluo-3 is assumed to be equal to that reported for the bathing solution, and may not reflect the actual value. Furthermore, Ca^2+^ binding kinetics for fluo-3 were taken from experiments performed *in vitro* (Naraghi 1997) and may not represent kinetics within an intact myocyte. Flux-clamp was developed on the assumption that most cytosolic Ca^2+^ fluxes would not be strongly affected by withdrawing fluo-3 and that the Ca^2+^ dependence of myofilament buffering and SERCA activity were adequately described by their respective model representations. This series of assumptions seems reasonable, however, as, through the use of the flux-clamp protocol, a set of myofilament parameters was determined that was able to describe the Ca^2+^ contraction dynamics of both mid- and endocardial cells. Furthermore, this parameter set displayed a steady-state force–Ca^2+^ relationship similar to those reported experimentally in isolated canine myocytes (Wolff *et al*. 1995).

## 5. Conclusion

We have used an integrative model of ventricular myocyte function that includes electrophysiology, Ca^2+^ handling and myofilament dynamics to explore potential mechanisms by which myocytes within the same heart modulate their EC coupling characteristics. Our work identifies *I*_to1_ and crossbridge cycling kinetics as primary mechanisms underlying transmural variation of myocyte contractile function.

## Supplementary Material

Additional data Coupling of EP/Ca^2+^-handling and myofilament models; ‘Flux-clamp’ simulation protocol; Testing hypothesized epicardial cell myofilament alterations
