# Supplementary material for: Mechanisms of transmurally varying myocyte electromechanics in an integrated computational model
Source: Philos Trans A Math Phys Eng Sci. 2008 Jul 1;366(1879):3361–80. doi: 10.1098/rsta.2008.0088 (PMC2556206; doi:10.1098/rsta.2008.0088)
Supplement: Additional data — Coupling of EP/Ca2+-handling and myofilament models; ‘Flux-clamp’ simulation protocol; Testing hypothesized epicardial cell myofilament alterations [file rsta20080088s01.pdf]

Supplemental Data

In this supplement, we summarize all modifications to published model parameters and equations that were completed in the course of this study. All equations and parameters not mentioned here are identical to those reported in the original publications (1-3). In some cases, we modified equations which are not contained specifically in these most recent reports and instead reference still earlier sources. In these instances, we have attempted to cite the earliest source in order to maintain a clear connection to past work.

Coupling of EP/Ca^{2+}-handling and myofilament models

The Greenstein canine myocyte EP/Ca^{2+}-handling model (1) as modified by Flaim and co-workers (2) was coupled to the Rice myofilament model (3) via the quantity $[Ca^{2+}]_i$. Tension and length-dependent feedback from the myofilaments was incorporated by replacing static buffering of Ca^{2+} by troponin C (TnC) as used in the original EP/Ca^{2+}-handling model with the dynamic buffering scheme formulated by Rice et al. (3). We replaced the right-hand side of equation A.84 in (4) with that of equation A50 in (3) to give the following:

$$\frac{d[LTRPNCa]}{dt} = [LTRPN]_{tot} \times \frac{d}{dt} Trop_{Apparent}(x) \qquad \text{(S1)}$$

With these modifications completed, we now refer to the combined models as the ‘fully-coupled’ model. Changes in parameter values for the fully-coupled model from those used originally are summarized in table S1 below.

Representing Ca^{2+} buffering by fluo-3

We represented the buffering of intracellular Ca^{2+} by fluo-3 dye by adding equation S2 below to the existing model. Buffering was assumed to follow standard kinetics, meaning that the rate of change of Ca^{2+} bound to fluo-3 can be straightforwardly written as:

$$\frac{d[Ca^{2+}]_{fluo}(t)}{dt} = k_{on}^{fluo}[Ca^{2+}]_i(t)\left([fluo]_{tot} - [Ca^{2+}]_{fluo}(t)\right) - k_{off}^{fluo}[Ca^{2+}]_{fluo}(t) \qquad \text{(S2)}$$

where $[Ca^{2+}]_{fluo}$ is the concentration of Ca^{2+} bound to the indicator, $[Ca^{2+}]_i$ is the concentration of free cytosolic Ca^{2+}, $[fluo]_{tot}$ is the total concentration of the indicator, and k_{on}^{fluo} and k_{off}^{fluo} are kinetic rate constants. Constants used throughout this study for fluo-3 buffering are listed in table S2 below. Fluo-3 kinetic parameters were taken from the work of Naraghi (5), who measured these values at 22 degrees C.

The Ca^{2+} flux associated with buffering by fluo-3, J_{fluo}, was set equal to equation S2 and added to equation A.87 from (4) as follows:

$$\frac{d[Ca^{2+}]_i}{dt} = \beta_i \left\{ J_{xfer} - J_{up} - J_{trpn} - J_{fluo} - \left(I_{Ca,b} - 2I_{NaCa} + I_{p(Ca)}\right)\frac{A_{cap}C_{sc}}{V_{myo}F} \right\} \qquad \text{(S3)}$$

'Flux-clamp' simulation protocol

Motivation for the flux-clamp is described in the main text, while the details of its implementation are presented here. In order to drive the myofilament model with a generic Ca^{2+} flux rather than a fixed Ca^{2+} transient, a simplified version of equation S3 was added to the myofilament model of Rice et al. (3). All Ca^{2+} fluxes except those corresponding to buffering by fluo-3, buffering by the low-affinity regulatory sites of TnC, and Ca^{2+} uptake by SERCA were lumped into a single, generic flux term J_{clamp}. Equation S3 now becomes

$$\frac{d[Ca^{2+}]_i}{dt} = \beta_i \left(J_{clamp} - J_{up} - J_{trpn} - J_{fluo} \right) \qquad \text{(S4)}$$

where J_{fluo} is as defined above. Ca^{2+} uptake by SERCA is approximated using the formulation of Shannon et al. (6) with parameters as defined by Winslow et al. (table 4 in (4)). As the myofilament model does not calculate the concentration of Ca^{2+} in the SR, $[Ca^{2+}]_{SR}$ is assumed have a constant value of 0.55 mM (this value corresponds to an average SR load produced by the electromechanics model using ENDO parameters and a BCL of 2000 ms). The quantity J_{trpn} is set equal to equation S1. Lastly, β_i represents the scaling coefficient of the rapid buffering approximation for calmodulin, with identical parameters and formulation to that used by Winslow et al. (4).

Representative time courses of fluo-3/Ca^{2+} fluorescence for epi-, mid-, and endocardial cells were digitized from (7) and normalised. Each point was then converted to approximate $[Ca^{2+}]_{fluo}$ using the following formula:

$$[Ca^{2+}]_{fluo} = \left([Ca^{2+}]_{fluo}^{\max} - [Ca^{2+}]_{fluo}^{\min} \right) y + [Ca^{2+}]_{fluo}^{\min} \qquad \text{(S5)}$$

where

$$[Ca^{2+}]_{fluo}^{\max} = \frac{[Ca^{2+}]_i^{\max} [fluo]_{tot}}{\frac{k_{off}}{k_{on}} + [Ca^{2+}]_i^{\max}} \qquad \text{(S6)}$$

and

$$[Ca^{2+}]_{fluo}^{\min} = \frac{[Ca^{2+}]_i^{\min} [fluo]_{tot}}{\frac{k_{off}}{k_{on}} + [Ca^{2+}]_i^{\min}} \qquad \text{(S7)}$$

The quantities k_{on} and k_{off} are those reported in table S2. $[Ca^{2+}]_i^{max}$ was assumed to be the mean Ca^{2+} transient magnitude reported for each respective cell type in (7), while $[Ca^{2+}]_i^{min}$ was assumed to be 0.1 µM.

The generic Ca^{2+} flux, J_{clamp}, was initialized as a piece-wise linear function in time and adjusted via non-linear least-squares solver (lsqnonlin, MATLAB) until the

modelled time course of $[Ca^{2+}]_{fluo}$ closely fit that extracted from the literature for the appropriate cell type. After fitting, the concentration of fluo-3 was set to zero, and J_{clamp} was used to elicit a cell shortening event. Myofilament model parameters were adjusted such that after fitting J_{clamp} to the midmyocardial cell Ca^{2+} transient and removing fluo-3, the model predicted unloaded shortening similar to measurements from (7). Final model parameters are reported in table S3 below in the column marked BASE.

Testing hypothesized epicardial cell myofilament alterations

We proposed several potential mechanisms for explaining altered Ca^{2+}-contraction displayed by epicardial cells. These hypotheses were tested by altering myofilament model parameters in suitable ways and repeating determination of J_{clamp} via the flux-clamp protocol. Table S3 contains parameter sets used in each case, labelled according to their abbreviations in the main text.

Table S1. Combined model of cellular electromechanics: Parameters Altered from Originals

Parameter	Definition	Source	Original Value	Modified Value	Units
NumCaRU	Number of Ca^{2+} release units	(1)	50000	75000	-
n_{perm}	Hill coeff.-like modifier of steepness of cooperative myofilament activation	(3)	15	7.5	-
$perm_{50}$	Approx. half-activation value of myofilaments	(3)	0.5	0.55	-
xbmodsp	Species-dependent XB-cycling kinetics scaling coeff.	(3)	1.0 (rat)	0.2 (rabbit)	-
mass	Mass coeff. for 1-D cell mechanics	(3)	5.00E-05	2.00E-05	norm. force s^2 μm^{-1}

Table S2. Fluo-3 kinetic parameters, from Naraghi (1997)

Parameter	Definition	Value
k_{off}^{fluo}	Rate of Ca^{2+} dissociation from fluo-3	0.369 ms^{-1}
k_{on}^{fluo}	Binding rate of Ca^{2+} to fluo-3	0.71 μM^{-1} s^{-1}

Table S3. Myofilament model parameters used in flux-clamp simulations

Parameter Name	Original Value	Parameter set (see main text): BASE	V1	TITIN	MyBPC	SENS	COOP	TITIN+COOP	Units
n_{perm}	15	9	-	-	-	8	11	11	unitless
$perm_{50}$	0.5	0.85	-	-	-	0.75	0.775	0.775	unitless
xbmodsp	1	0.2	1	-	-	-	-	-	unitless
k_{on}	0.05	0.1	-	-	-	-	-	-	$\mu M^{-1}s^{-1}$
$PCon_{titin}$	0.002	0.002	-	0.006	-	-	-	0.006	norm. force
$PExp_{titin}$	10	10	-	30	-	-	-	30	unitless
Temp	310	312	-	-	-	-	-	-	K
f_{app}	0.5	0.5	-	-	1	-	-	-	ms^{-1}

Values not displayed are identical to those of BASE

Literature Cited

1. Greenstein, J. L., R. Hinch and R.L. Winslow 2006. Mechanisms of excitation-contraction coupling in an integrative model of the cardiac ventricular myocyte. Biophys. J. 90, 77-91.

2. Flaim, S. N., W.R. Giles and A.D. McCulloch 2006. Contributions of sustained INa and IKv43 to transmural heterogeneity of early repolarization and arrhythmogenesis in canine left ventricular myocytes. Am. J. Physiol. Heart Circ. Physiol. 291, H2617-29.

3. Rice, J. J., F. Wang, D.M. Bers and P.P. de Tombe 2008. Approximate model of cooperative activation and crossbridge cycling in cardiac muscle using ordinary differential equations. Biophys. J.

4. Winslow, R. L., J. Rice, S. Jafri, E. Marban and B. O'Rourke 1999. Mechanisms of altered excitation-contraction coupling in canine tachycardia-induced heart failure, II: Model studies. Circ. Res. 84, 571-586.

5. Naraghi, M. 1997. T-jump study of calcium binding kinetics of calcium chelators. Cell Calcium. 22, 255-268.

6. Shannon, T. R., K.S. Ginsburg and D.M. Bers 1998. Reverse mode of the sarcoplasmic reticulum ca pump limits sarcoplasmic reticulum ca uptake in permeabilized and voltage-clamped myocytes. Ann. N. Y. Acad. Sci. 853, 350-352.

7. Cordeiro, J. M., L. Greene, C. Heilmann, D. Antzelevitch and C. Antzelevitch 2004. Transmural heterogeneity of calcium activity and mechanical function in the canine left ventricle. Am. J. Physiol. Heart Circ. Physiol. 286, H1471-9.
